# 阿帕替尼用于小细胞肺癌多线治疗后的挽救治疗

**DOI:** 10.3779/j.issn.1009-3419.2018.07.11

**Published:** 2018-07-20

**Authors:** 芳芳 李, 海涛 陶

**Affiliations:** 1 100853 北京，中国人民解放军总医院健康管理研究院国宾部 Department of State Guest, Institute of Health Management, the Chinese PLA General Hospital, Beijing 100853, China; 2 100853 北京，中国人民解放军总医院 肿瘤内科 Department of Oncology, the Chinese PLA General Hospital, Beijing 100853, China

**Keywords:** 小细胞肺癌, 抗血管生成, 阿帕替尼, Gilbert综合征, Small cell lung cancer, Angiogenesis, Apatinib, Gilbert syndrome

## Abstract

小细胞肺癌（small cell lung cancer, SCLC）恶性程度高，在放、化疗失败后缺乏有效治疗手段，抗血管生成治疗在晚期SCLC中表现出一定的疗效。阿帕替尼是一种口服小分子酪氨酸激酶抑制剂，通过抑制血管内皮生长因子受体2（vascular endothelial growth factor receptor 2, VEGFR-2）达到抗血管生成的作用，在SCLC上的应用报道较少。本文报道了1例合并有Gilbert综合征的SCLC患者，在4线化疗失败后接受了阿帕替尼的挽救治疗，1个月后达到部分缓解（partial remission, PR），最终获得了5个月的无进展生存时间。由于胆红素代谢障碍，该患者在阿帕替尼治疗过程中反复出现3级的高胆红素血症，余不良反应耐受良好。阿帕替尼在晚期SCLC挽救治疗中的作用值得进一步探索。

## Abstract (en)

Small cell lung cancer (SCLC) was highly malignant and lack effective treatment after the failure of radiotherapy and chemotherapy. Antiangiogenic therapy had shown a certain effect in advanced SCLC. Apatinib, a new potent oral small-molecule tyrosine kinase inhibitor targeting the intracellular domain of vascular endothelial growth factor receptor 2 (VEGFR-2), showed the effect of anti-angiogenesis. However, the efficacy in SCLC was rarely reported. We reported 1 case of advanced SCLC with Gilbert syndrome, the patient received Apatinib after the failure of 4 lines of chemotherapy, and achieved a partial response according to the Response Evaluation Criteria in Solid Tumors (RECIST) 1.1 standard after one month. The progression-free survival (PFS) was 5 months. Apatinib was well tolerated except recurrent grade 3 hyperbilirubinemia because of the metabolic disorder of Bilirubin. Salvage treatment with Apatinib for advanced SCLC deserved further exploration.

小细胞肺癌（small cell lung cancer, SCLC）占所有肺癌的15%-20%，具有恶性程度高、倍增时间快、侵袭性强、预后差的特点^[1]^。在初次诊断时，约60%的SCLC存在远处转移，仅有5%的患者能够接受手术治疗，绝大多数患者目前主要治疗手段是化疗和放疗。尽管SCLC对初识的放、化疗较为敏感，但一旦出现耐药后续的治疗选择较少。80%的SCLC存在血管内皮生长因子（vascular endothelial growth factor, VEGF）的表达，在SCLC上也开展了很多抗血管生成类药物治疗的临床研究，但多数研究未能取得突破性进展^[2, 3]^。阿帕替尼是我国自主研发的血管内皮生长因子受体2（vascular endothelial growth factor receptor 2, VEGFR-2）的抑制剂，在胃癌、乳腺癌等多种实体瘤中有不错的疗效，但在SCLC上应用报道较少。本院最近收治了1例SCLC患者，在四线化疗失败后接受了抗血管生成药物阿帕替尼单药的治疗，达到部分缓解的疗效，获得了5个月的无进展生存期（progression free survival, PFS），现报道如下。

## 1 病例资料

患者男性，62岁，因“刺激性干咳2月余”入院。患者于2014年1月初无明显诱因出现咳嗽，以刺激性干咳为主，无痰，予以对症止咳治疗，效果欠佳。2014年3月触及锁骨上淋巴结肿大，当地医院行胸部计算机断层扫描（computed tomography, CT）示纵隔内及右肺门可见多发肿大淋巴结影，部分融合成块。锁骨上淋巴结切除活检示：小细胞癌，肺来源可能性大，免疫组化：CD56（+++）、CgA（-）、Syn（+++）、ki-67（60%+）、LCA（-）、CD21（-）、TIF-1（+++）、NapsinA（-）、CEA（-）、CK7（-）、CK20（个别阳性）、CK8/18（-）、CD5（-）、c-kit（-）、TDT（-）。正电子发射断层显像（positron emission tomography computed tomography, PET-CT）示：双侧锁骨上窝、纵隔、右肺门多发高代谢淋巴结; 全身骨多发代谢增高，考虑髓内受累，请结合骨穿结果。后于2014年4月1日行右髂骨骨髓活检，病理示：异常细胞呈弥漫性浸润造血基质，结合临床，考虑肿瘤细胞骨髓浸润。故患者明确为右肺小细胞癌（TxN3M1，Ⅳ期）。既往有吸烟史40年，每日约20支，确诊肺癌后戒烟。患者自50岁单位年度体检多次发现轻度总胆红素素水平（25 μmol/L-30 μmol/L）及非结合胆红素水平升高（20 μmol/L-25 μmol/L），相关影像学检查及其他检验未见溶血性疾病证据，临床诊断考虑为Gilbert综合征。

综合患者情况，一线给予EC（依托泊苷联合卡铂）方案化疗，2014年4月-8月共行6个周期治疗，2个周期后疗效评估为部分缓解（partial remission, PR），4个周期（2014年6月）后复查PET-CT示双侧锁骨上窝、纵隔、右肺门、骨多发高代谢灶基本消失，肿瘤标记物正常，疗效评估为完全缓解（complete remission, CR）。患者于2014年7月患者开始接受预防性脑放疗（25 Gy/10 F），2014年8月开始接受胸部序贯放疗，靶区：肺部病灶及区域淋巴结（50.4 Gy/28 F）。一线放化疗耐受尚可，治疗中出现3级总胆红素升高（最高79.1 μmol/L）、2级脱发、1级消化道反应及3级白细胞降低。后定期复查，2015年7月入院复查右肺门病灶较前明显增大，余腹部CT、骨扫描未见明确新发病灶，疗效评估为疾病进展（progressive disease, PD）。2015年8月患者接受二线单药紫杉醇（白蛋白结合型）治疗1个周期，患者咳嗽症状明显进展，复查胸部CT示右肺门病灶较前明显进展，疗效评估为PD。患者行外周血UGT1A1检测示：UGT1A1^*^6 G/G野生型、UGT1A1^*^28 TA7/TA7纯合型。2015年8月-12月，患者接受6个周期IP方案（伊立替康联合顺铂）三线治疗，最佳疗效评估为PR。三线治疗期间经放疗科评估，无法再次胸部放疗。2016年8月复查胸部CT提示右肺门及纵隔病灶较前明显增大，疗效评估为PD。2016年8月-12月，患者接受6个周期EP（依托泊苷联合顺铂）方案四线化疗，最佳疗效评估为PR。

2017年4月入院复查胸部CT见右肺门病灶伴阻塞性改变，纵隔淋巴结转移，范围较前增大，疗效评估为PD。因考虑患者既往接受多线化疗及放疗，美国东部肿瘤协作组（Eastern Cooperative Oncology Group, ECOG）评分为1分，可选治疗方案有限，建议患者口服阿帕替尼治疗。4月15日开始口服阿帕替尼治疗，起始剂量为每日250 mg，7 d后剂量增加至500 mg，服用5 d左右患者出现声音嘶哑，并逐渐出现巩膜轻度黄染，7 d后增量至750 mg，加量后第3天后患者出现全身皮肤、巩膜黄染明显加重，声音嘶哑加重，胸前区少量红色皮疹，脚趾、耳部出现多个角质层增厚小结节。相关检验示：总胆红素升高121.6 μmol/L，分级3级，直接胆红素4.5 μmol/L，丙氨酸氨基转移酶正常，考虑阿帕替尼所致高胆红素血症，给予丁二磺酸腺苷蛋氨酸治疗，7 d后复查总胆红素降至73.3 μmol/L，声音嘶哑较前有所恢复，咳嗽临床症状较前明显减轻。患者为单纯的高胆红素血症不合并有转氨酶升高，且声音嘶哑等不良反应好转，咳嗽等肿瘤导致的临床症状有所缓解，判断患者对阿帕替尼耐受较好。患者高胆红素血症不除外与Gilbert综合征本身的胆红素代谢障碍有关，故决定继续给予阿帕替尼治疗。根据既往SCLC的诊疗经验^[4]^，于5月9日继续给予继续口服阿帕替尼250 mg治疗。5月26日复查胸部CT见右肺门肺癌伴阻塞性改变、纵隔淋巴结转移，较前缩小，疗效评估为PR（图 1）。患者继续口服阿帕替尼250 mg治疗，该周期患者出现胆红素升高3级（70 μmol/L-106 μmol/L），声音嘶哑1级，手足综合征1级。7月11日复查胸部CT示右肺门肺癌伴阻塞性改变，纵隔淋巴结转移，右上肺动脉局部侵犯可能，较前相仿，综合疗效评估为疾病稳定（stable disease, SD）（图 1）。继续口服阿帕替尼250 mg治疗。8月14日复查，提示右肺门肺癌伴阻塞性改变，较前局部进展; 纵隔淋巴结转移，右上肺动脉局部侵犯可能，较前相仿，综合疗效评估为SD，但肿瘤已有进展表现（图 2）。患者无明显临床症状，遂将阿帕替尼剂量调整为250 mg/500 mg交替使用，治疗后出现高血压2级，高胆红素血症3级，声嘶1级，手足综合征1级。2017年9月入院复查，肺CT示右肺癌伴阻塞性改变，较前局部进展，综合疗效评估为PD（图 2）。而后患者更换阿帕替尼（250 mg，每天1次）联合替莫唑胺胶囊（150 mg/m^2^, d1-d5），患者口服2日替莫唑胺后出现腹部疼痛、不能进食后停止阿帕替尼及替莫唑胺治疗。2017年10月患者院外自行购买Nivolumab治疗，2017年12月随访仍生存，复查胆红素水平已逐步下降至正常值（2017-10-15，总胆红素20.5 μmol/L）。

**1:**
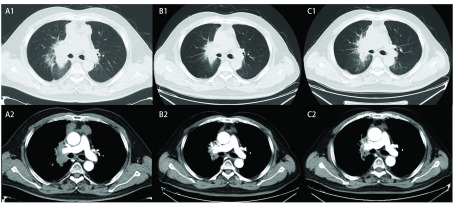
影像学评价（阿帕替尼治疗后第1、3个月）。 The response evaluation of Apatinib treatment after 1 month and 3 months.

**2:**
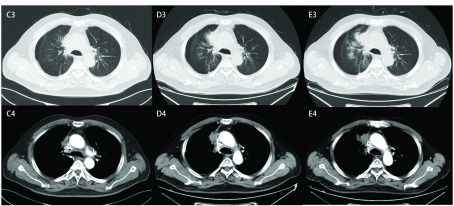
影像学评价（阿帕替尼治疗后第3、4、5个月）。 The response evaluation of Apatinib treatment after 3 months, 4 months and 5 months.

## 2 讨论

SCLC是恶性程度非常高的肿瘤，进展快，近30年来SCLC的治疗上几乎缺乏突破性的进展，对于广泛期SCLC而言，目前的中位生存期仍在9个月左右。虽然SCLC对一线化疗敏感度很高，但绝大多数患者很快复发，后续缺乏有效的治疗药物，所以急需探索对SCLC有效的后续治疗选择。

新生血管是恶性肿瘤进展、侵袭的重要基础，抗血管生成治疗在很多恶性肿瘤治疗上都取得一定突破，在SCLC中也开展了很多抗血管生成治疗的探索。VEGF的单抗贝伐珠单抗是应用最为广泛的抗血管生成药物，在SCLC中开展的早期两项单臂的2期临床研究CALGB 30306和E3501显示出较好的有效性和安全性（跟历史数据对比），随后开展的SALUTE、IFCT-0802等研究均提示联合贝伐珠单抗延长了PFS，但总生存期（overall survival, OS）获益不明显^[5-8]^。鉴于此，贝伐珠单抗并未批准用于SCLC的治疗，临床应用也较少。多靶点的抗血管生成药物舒尼替尼、凡德他尼、索拉非尼、帕唑替尼、阿柏西普（Aflibercept）等也在SCLC上进行了研究尝试。GALGB30504评估了舒尼替尼（150 mg达峰剂量后37.5 mg，*qd*）用于广泛期SCLC化疗后维持治疗的疗效，结果显示，维持治疗延长了患者PFS（2.1个月*vs* 3.7个月，*P*=0.02），但OS无获益^[9]^。阿柏西普在与拓扑替康联合治疗铂类耐药的SCLC患者中获得了3个月的PFS改善，但OS并没有明显改善且不良反应相应增加^[10]^。总体而言，抗血管生成类药物治疗在SCLC上能够延长部分患者的PFS，但未能延长OS，故未能常规应用于临床，该类药物在SCLC上的应用仍需进一步研究。

阿帕替尼是一种口服小分子酪氨酸激酶抑制剂，选择性地结合并抑制VEGFR-2，通过抗血管生成作用起到抗肿瘤作用，首先被批准用于晚期胃癌三线及三线以上方案时使用，同时在三阴性乳腺癌、非小细胞肺癌（non-small cell lung cancer, NSCLC）及晚期肝胆癌中进行2期临床研究，在多种实体瘤的治疗中显现出一定的疗效。阿帕替尼在SCLC的多线治疗上也进行了尝试。曲范杰等^[11]^报道了1例四线给予多西他赛联合阿帕替尼治疗的病例，该患者在治疗1个月后达到了PR的疗效，但很快因并发心率失常死亡。洪卫等^[4]^在2016年WCLC中首次报道了阿帕替尼治疗二线或三线化疗失败后广泛期SCLC的单中心回顾性研究。入组了13例广泛期SCLC患者，口服阿帕替尼500 mg，如出现3级及以上的不良反应则减量至250 mg，最终45.5%（5/11）的患者经历了减量，在可评估的11例患者中，疾病控制率为81.8%（9/11），其中PR者占18.2%（2/11），SD者占63.6%（7/11）。主要的3级或4级不良反应为高血压（27.3%, 3/11）、口腔粘膜炎（9.1%, 1/11）和乏力（9.1%, 1/11）。从该小样本的临床研究中可以观察阿帕替尼对于多线治疗后的SCLC有一定疗效，值得进一步的研究。

本次报道病例也是多重治疗之后的患者，既往对化疗较为敏感，接受过一线EC、二线单药紫杉醇（白蛋白结合型）、三线IP及四线EC方案治疗（图 3）。除二线治疗单药紫杉醇未获得肿瘤控制外均获得了PR的疗效，但从病程可以看出该患者化疗后控制时间越来越短，化疗耐药发生加速，且后续化疗耐受差，故多线治疗后给予阿帕替尼治疗，达到了PR的疗效，获得了5个月的PFS时间。阿帕替尼作为多线治疗后的挽救方案，有一定疗效。从现有的2例有详细病史的病例来看，2个病例具有下列相同的特点：既往对化疗药物敏感（一线、二线、三线治疗均达到SD或以上）、未发生脏器转移进展（因肺原发灶或淋巴结增大进展）、患者体力状况好（ECOG 2分以上）等特点，提示我们，对于临床体力状况良好又缺乏有效治疗手段的患者，阿帕替尼也可以作为一种临床选择，但目前相关证据不足，抗血管生成药物出血、高血压等风险较大，应用时需充分权衡获益及风险。

**3:**
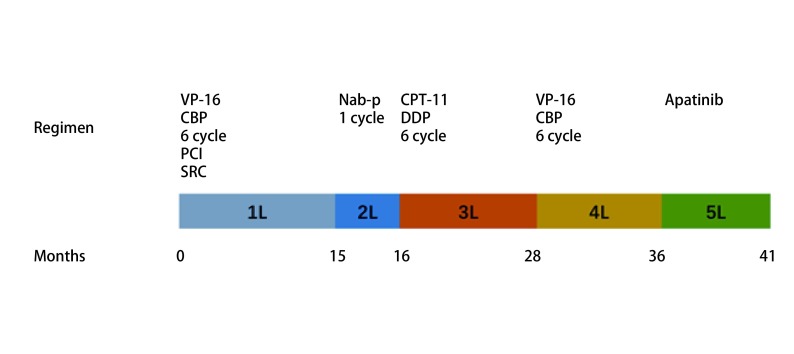
治疗时间轴。 Timeline of patient's management.

从前述的回顾性临床研究及本例报道可以发现阿帕替尼的不良反应发生率较高。前述研究中，患者中口服500 mg剂量的阿帕替尼仍有45.5%的患者发生了药物减量。本例患者在早期服药增量至750 mg时出现了严重的高胆红素血症，后期经过降黄及剂量调整后胆红素仍维持于2级-3级不良反应水平。虽然进行了剂量减低（250 mg），该患者在后期还出现了声音嘶哑、手足综合征、高血压等不良反应。虽然后期不良反应临床可耐受，但前期的药物减量势必影响疗效，在减量后的第3个月肿瘤已出现进展趋势。从NSCLC以及SCLC的应用经验来看（500 mg剂量为主），肺癌患者对阿帕替尼的耐受明显差于胃癌患者（批准剂量850 mg），提示不同瘤种对阿帕替尼的耐受存在差异，后续对于肺癌患者应用剂量应该个体化，250 mg的起始剂量也是临床选择^[12]^。

该例患者既往体检多次发现轻度的总胆红素血症，以非结合胆红素升高为主，且UGT1A1^*^28基因型为TA7/TA7，符合Gilbert综合征的临床表现和发病机制，故诊断为Gilbert综合征。Gilbert综合征是一种较常见的遗传性非结合胆红素血症，以非结合胆红素升高为主。患者的肝细胞器微粒体中胆红素葡萄糖醛酸转移酶活力不足，影响非结合胆红素在肝细胞内结合反应的正常进行，致使肝细胞对胆红素的摄取也受到障碍，而造成肝细胞对非结合型胆红素的摄取和结合功能的双缺陷^[13]^。该患者在化疗时也出现高胆红素血症，多为1级-2级，未进行剂量调整，可能与Gilbert综合征导致的胆红素代谢障碍有关。阿帕替尼的同类药物培唑帕尼也可能使血清总胆红素水平升高。在236例高加索患者的合并药物基因分析发现UGT1A1^*^28 TA7/TA7纯合型应用培唑帕尼后发生高胆红素血症的几率相对于UGT1A1^*^28 TA6/TA6和TA6/TA7基因型显著增加^[14]^。该患者为UGT1A1^*^28 7/7纯合型，在治疗中也出现3级高胆红素血症，经剂量调整及降黄治疗后好转。目前关于阿帕替尼的药物代谢研究并未发现UGT1A1与阿帕替尼的代谢存在相关性^[15]^。但本病例提示UGT1A1可能与阿帕替尼的代谢相关，不同基因型可能影响药物代谢病对后期疗效和不良反应发生产生影响，值得进一步探索。

综上所述，阿帕替尼可作为SCLC多线治疗后的选择，但需密切注意和控制不良反应，关于阿帕替尼可能的获益人群需要进一步的研究探索。

## References

[1] Liu B, Qin JW, Zhou JM (2017). Advances in the treatment of relapsed small cell lung cancer. Zhongguo Fei Ai Za Zhi.

[2] Dowell JE, Amirkhan RH, Lai WS (2004). Survival in small cell lung cancer is independent of tumor expression of VEGF and COX-2. Anticancer Res.

[3] Tan WL, Jain A, Takano A (2016). Novel therapeutic targets on the horizon for lung cancer. Lancet Oncol.

[4] Hong W, Li H, Jin X (2017). P1.07-053 apatinib for chemotherapy-refractory extensive stage SCLC: results from a single-center retrospective study. J Thorac Oncol.

[5] Horn L, Dahlberg SE, Sandler AB (2009). Phase Ⅱ study of cisplatin plus etoposide and bevacizumab for previously untreated, extensive-stage small-cell lung cancer: Eastern Cooperative Oncology Group Study E3501. J Clin Oncol.

[6] Ready NE, Dudek AZ, Pang HH (2011). Cisplatin, irinotecan, and bevacizumab for untreated extensive-stage small-cell lung cancer: CALGB 30306, a phase Ⅱ study. J Clin Oncol.

[7] Tiseo M, Boni L, Ambrosio F (2017). Italian, multicenter, phase Ⅲ, randomized study of cisplatin plus etoposide with or without bevacizumab as first-line treatment in extensive-disease small-cell lung cancer: The GOIRC-AIFA FARM6PMFJM Trial. J Clin Oncol.

[8] Spigel DR, Townley PM, Waterhouse DM (2011). Randomized phase Ⅱ study of bevacizumab in combination with chemotherapy in previously untreated extensive-stage small-cell lung cancer: Results from the SALUTE Trial. J Clin Oncol.

[9] Ready NE, Pang HH, Gu L (2015). Chemotherapy with or without maintenance sunitinib for untreated extensive-stage small-cell lung cancer: A randomized, double-blind, placebo-controlled phase Ⅱ study-CALGB 30504 (Alliance). J Clin Oncol.

[10] Allen JW, Moon J, Redman M (2014). Southwest Oncology Group S0802: A randomized, phase Ⅱ trial of weekly topotecanwith and without ziv-aflibercept in patients with platinum-treated small-cell lung cancer. J Clin Oncol.

[11] Qu FJ, Yu WW, Zhang J (2017). A case report and literature review of apatinib combined with chemotherapy for treatment on advanced small cell lung cancer. Zhongguo Zhong Liu Sheng Wu Zhi Liao Za Zhi.

[12] Song Z, Yu X, Lou G (2017). Salvage treatment with apatinib for advanced non-small-cell lung cancer. Onco Targets Ther.

[13] Song JY, Sun M, Li JY (2016). Role of *UGT1A1* gene polymorphism in the pathogenesis of gilbert syndrome. Lin Chuang Gan Dan Bing Za Zhi.

[14] Xu CF, Reck BH, Xue Z (2010). Pazopanib-induced hyperbilirubinemia is associated with Gilbert's syndrome *UGT1A1* polymorphism. Br J Cancer.

[15] Ding J, Chen X, Gao Z (2013). Metabolism and pharmacokinetics of novel selective vascular endothelial growth factor receptor-2 inhibitor apatinib in humans. Drug Metab Dispos.

